# Effects of synthetic colloid and crystalloid solutions on hemorheology *in vitro* and in hemorrhagic shock

**DOI:** 10.1186/s40001-015-0088-6

**Published:** 2015-02-04

**Authors:** Gan Chen, Jingxiang Zhao, Penglong Li, Xuemei Kan, Guoxing You, Ying Wang, Yujing Yin, Xin Luo, Yuhua Zhang, Lian Zhao, Hong Zhou

**Affiliations:** Institute of Transfusion Medicine, Academy of Military Medical Sciences, No. 27th Taiping Road, HaiDian, Beijing China

**Keywords:** Plasma expander, Synthetic colloids, Hemorrhagic shock, Plasma viscosity, Erythrocyte aggregation

## Abstract

**Background:**

Plasma expanders are commonly used in the management of critically ill patients, which may exhibit altered hemorheology. We evaluated the effects of various synthetic colloids and Lactated Ringer’s (LR) solution on hemorheological parameters *in vitro* and in a rodent hemorrhagic shock model.

**Methods:**

For the *in vitro* experiments, rat blood was incubated with hydroxyethyl starch (HES) 130/0.4, HES 200/0.5, succinylated gelatine (GEL), or LR at various ratios. The control consisted of blood without dilution. The hemorheological parameters were measured after a 15-min incubation. For the *in vivo* study, rats were subjected to a severe volume-controlled hemorrhage and were resuscitated using a colloid solution (HES 130/0.4, HES 200/0.5, or GEL) or LR. The hemorheological parameters were measured 2 h after resuscitation.

**Results:**

The GEL significantly elevated the plasma viscosity compared to the other groups. In the *in vitro* study, GEL and LR accelerated the erythrocyte aggregation. There was no significant difference between HES 130/0.4, HES 200/0.5, and control groups regarding the aggregation amplitude and index. In the *in vivo* study, the aggregation amplitude increased significantly in the GEL group compared to the HES 130/0.4, HES 200/0.5, LR, and sham groups. There was no significant difference between the groups with respect to the elongation index *in vivo*.

**Conclusions:**

Hydroxyethyl starch did not change the erythrocyte aggregation compared to the control. GEL significantly accelerates the erythrocyte aggregation and elevates the plasma viscosity compared to hydroxyethyl starch. The *in vitro* hemorheological measurements most likely provide hints for the *in vivo* study.

## Background

The administration of plasma expanders is crucial for managing critically ill patients across a range of clinical conditions, including major surgery, hemorrhagic shock (HS), and trauma [1]. There is growing evidence that plasma expanders, which are often given in large volumes during fluid therapy, are associated with altered hemorheological parameters, which play an important role in modulating microcirculatory and tissue oxygenation [2,3]. Previous studies have demonstrated the effects of various fluids on hemorheology [4-7]. Dextran increased the plasma viscosity and induced erythrocyte aggregation by forming dextran bridges between the cells [8,9]. Hydroxyethyl starch (HES) 450/0.7 infusion elevated blood viscosity and erythrocyte aggregation while decreasing the erythrocyte deformability [6]. Gelatin was also found to increase erythrocyte aggregation [10]. However, direct comparisons of the hemorheological effects between plasma expanders, such as succinylated gelatin, HES 130/0.4, HES 200/0.5, and Lactated Ringer’s (LR) solution, are lacking. Among the hemorheological parameters, erythrocyte aggregation may be associated with tissue perfusion [11,12]. However, the effects of HES on erythrocyte aggregation remain controversial [4]. HES 200/0.5 and HES 130/0.4 were indicated to induce erythrocyte aggregation in both *in vitro* and *in vivo* studies [6,13,14]. However, other studies have shown that erythrocyte aggregation was unchanged in the presence of HES 200 compared to that observed in the control group [4,15].

The rheological measurements can be investigated *in vitro* and *in vivo. In vitro* techniques could permit screening of the rheological properties of different plasma expanders, such as hemoglobin-based oxygen carriers, by mixing erythrocytes and expanders in different ratios [16]. Moreover, the *in vitro* rheological effects of different solutions can be examined in normal and septic blood to predict further compromise *in vivo* [6]. *In vitro* rheological measurements show advantage in saving time and animal models. However, the *in vitro* rheological effects may differ significantly from the *in vivo* effects. The extent to which *in vitro* findings for hemorheological parameters are of relevancy for the *in vivo* situation needs to be further investigated.

Thus, to evaluate the effects of synthetic colloids and LR solution on hemorheology and the relationship between *in vitro* and *in viv*o rheological measurements, we compared HES 130/0.4, HES 200/0.5, succinylated gelatin, and LR to demonstrate their relative effects on hemorheology *in vitro*. Next, we verified the *in vitro* results using a rodent HS model *in vivo*. The hemorheological parameters include plasma viscosity, erythrocyte aggregation, and erythrocyte deformability.

## Methods

### Animal preparation

This study was approved by the ethics committee of the Institute of Transfusion Medicine, Academy of Military Medical Sciences. All efforts were made to minimize the number of animals used and their suffering. Fourty-eight male Wistar rats weighing 210–250 g (Vital River Laboratories, Beijing, China) were included in the study; 39 rats were used for the *in vivo* experiment. The remaining rats were used for the *in vitro* experiment. Animals were used after a minimum 5- to 7-day acclimation period.

The rats were anesthetized using sodium pentobarbital (50 mg/kg), which were injected intraperitoneally. Rats were allowed to breathe room air spontaneously in a supine position for the duration of the experiment. Body temperature was maintained at 37°C ± 0.1°C with the aid of a heating pad (Softron Beijing, Inc., TMS-201, China). The right femoral artery and vein were isolated with minimal dissection and catheterized using polyethylene catheters (PE-50) containing heparinized saline. Rats were heparinized with 400 U/kg heparin (IV). Supplementary doses of pentobarbital were administered when necessary.

### Blood sampling and treatment for the *in vitro* study

Fresh blood samples were obtained from the right femoral arterial catheter using heparin-treated tubes (15 U/mL). The rat blood was centrifuged for 5 min at 1,500 × g and adjusted to a hematocrit of 40% by adding or removing autologous plasma. To represent the clinically relevant colloid plasma peak levels reported in the literatures [9,17,18], the well-distributed erythrocyte samples were subsequently diluted with 6% HES 130/0.4 (HES 130) (Voluven®, Fresenius-Kabi, Bad Homburg, Germany), 6% HES 200/0.5 (HES 200) (HAES-steril®, Fresenius-Kabi, Beijing, China), 4% succinylated gelatin (GEL, 30 kDa) (Gelofusine®, B. Braun, Shenyang, China), or LR (Juneng, Siping, China); the volume ratios of blood with the test solutions are 5:1 and 3:1. The control group consisted of the erythrocyte suspension without dilution.

All samples were incubated for 15 min at 37°C, and then, blood samples were adjusted to a hematocrit of 40% before measuring the hemorheological parameters. The plasma was obtained using centrifugation to detect the viscosity. All rheological measurements were carried out at a temperature of 37°C, which are in accordance with the international guidelines for the measurement of hemorheological parameters [19].

### Hemorrhagic shock/resuscitation protocol for the *in vivo* study

A rat HS model was prepared as described previously [20]. After surgical preparation and 10 min of stabilization, a volume-controlled hemorrhage of 18 mL/kg was performed using pumps (Softron Beijing, Inc., Beijing, China) for 30 min through the right femoral arterial catheter. Rats were then subjected to a slower hemorrhage of 12–15 mL/kg for 35 min. Due to the individual differences of animals, the blood withdrawal rate changed with a certain range. The animals with a base excess of −9 to −12 mmol/L were resuscitated after blood withdrawal via the femoral venous catheter. The results of arterial blood gas analysis have been published in our previous study [20].

After the volume-controlled hemorrhage was induced, the animals were randomly assigned to one of five groups. The sham group underwent all instrumentation procedures without blood collection (*n* = 7). The other four groups each received a different colloid solution (HES 130, HES 200, GEL) or LR (*n* = 8/group). The volume of resuscitation for the colloid solutions was equal to that of the blood withdrawal and three times that of the blood withdrawal for LR; all the infusions were performed using a pump-driven constant infusion at a rate of 0.33 mL/min. After 2 h had elapsed following resuscitation, blood samples were collected from the femoral artery with heparin-treated tubes for hemorheological evaluation (15 U/mL). The animals were then euthanized by exsanguination under anesthesia.

### Hemorheological evaluation

Hemorheology was assessed through the plasma viscosity, erythrocyte deformability (elongation index (EI)), erythrocyte aggregation index (AI), and aggregation amplitude (AMP).

The plasma viscosity was measured using a capillary viscometer at 37°C (LBY-N6B, Precil Company, Beijing, China). The measurement of viscosity was based on analyzing the capillary pressure-driven movement of plasma whose mean velocity and, therefore, shear rate varies with time.

EI was measured at shear rates of 100, 400, 600, 800, and 1,000 s^−1^ using an ektacytometer based on laser diffraction at 37°C (LBY-BX, Precil Company, Beijing, China). For this purpose, a 40-μL blood sample was suspended in 1 mL of a highly viscous solution of polyvinylpyrrolidone (PVP, MW = 30 kDa) in an isotonic phosphate buffer base (pH 7.4). The EI was obtained based on laser diffraction patterns at user-defined shear stress values.

For the erythrocyte aggregation measurement, the ektacytometer (LBY-BX, Precil Company, Beijing, China) was employed. The measurement was based on the change in back-scattered light on abrupt cessation of the erythrocyte suspension (600 to 0 s^−1^) at 37°C [5,21]. After mixing 500-μL blood and 100-μL PVP buffer as described above, a 500-μL mixture was used to measure the aggregation parameters, including AI and AMP. Hyperaggregation is indicated by increased AI and AMP.

### Statistical analysis

Data are expressed as the mean ± standard deviation (SD) and were examined for normal distribution and homogeneity of variance using the Shapiro-Wilk test and Levene’s test. The means of each group were compared using one-way analysis of variance (ANOVA) followed by the Student-Newman-Keuls test when the normality and homogeneity of variance assumptions were satisfied; otherwise, ANOVA followed by Student-Newman-Keuls multiple range test was applied. *P* < 0.05 was considered significant.

## Results

### Plasma viscosity

**Figure 1:**
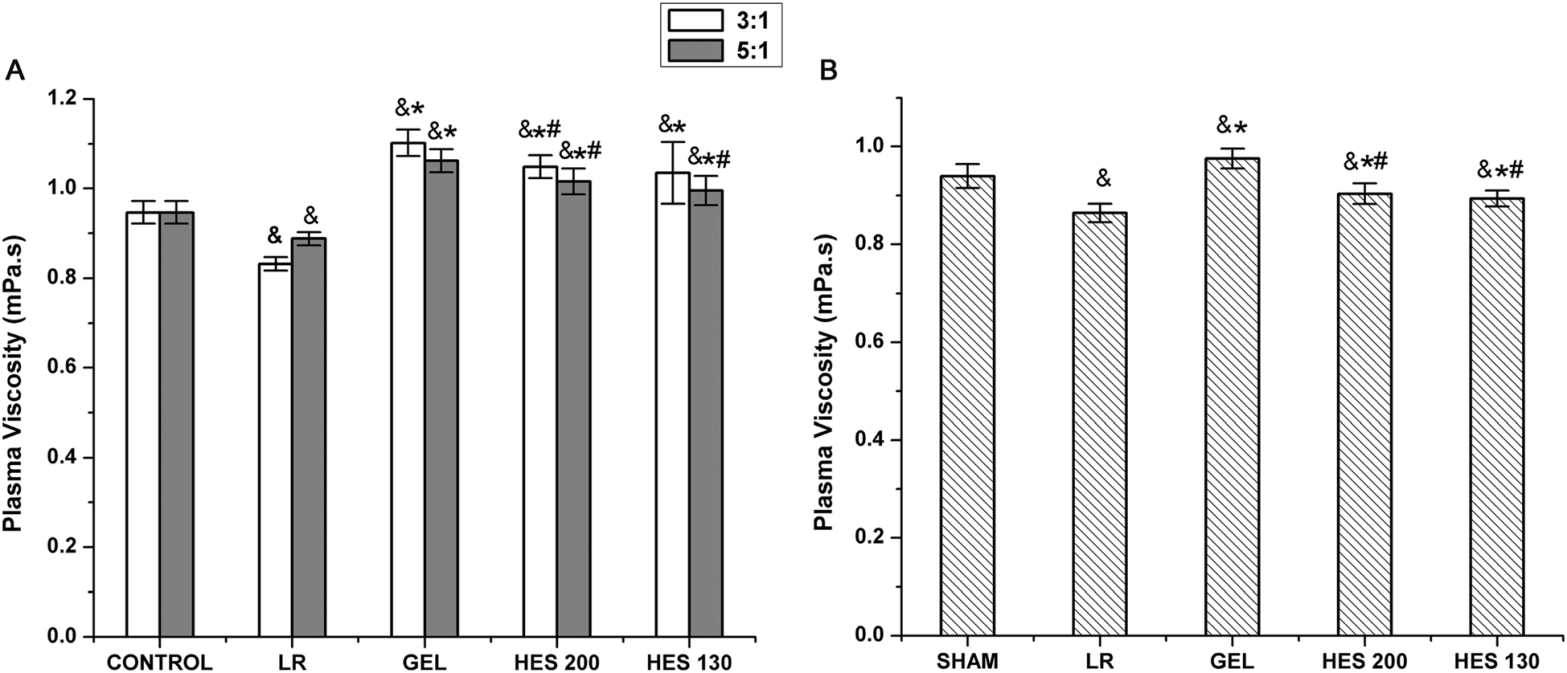
**Effects of various plasma expanders on the plasma viscosity. (A)** Plasma viscosity at various mixing ratios *in vitro* (*n* = 5). **(B)** Plasma viscosity *in vivo*. Data are plotted as the means ± SD. ^&^ *P* < 0.05 versus the sham or control group; *P < 0.05 versus the LR group; ^#^ *P* < 0.05 versus the GEL group.

The plasma viscosity measurements *in vitro* are presented in Figure 1A. The plasma viscosity was significantly increased in the presence of GEL, HES 130, and HES 200 compared to that of the control group (*P* < 0.05). The plasma viscosity levels were significantly higher in the GEL, HES 130, and HES 200 groups compared to that in the LR group (*P* < 0.05). The plasma viscosity in the HES 200 group was lower than that in the GEL group (*P* < 0.05). Moreover, there was no significant difference between the values in the HES 130 and HES 200 groups.

Plasma viscosity increased significantly after the infusion of GEL (*P* < 0.05) and decreased significantly after the infusion of HES 130, HES 200, and LR compared to the sham group *in vivo* (*P* < 0.05, Figure 1B). The plasma viscosity levels were significantly higher in the GEL, HES 130, and HES 200 groups compared to that in the LR group (*P* < 0.05). There was no significant difference between the values in the HES 130 and HES 200 groups.

To confirm whether the alterations in plasma viscosity were the result of a direct effect of the intrinsic viscosities of the infused solutions, we performed viscosity measurements of the plasma expanders. The viscosities increased in the following order: LR < GEL < HES 130 < HES 200 (Table 1).

**Table 1:** **Intrinsic viscosity of the four plasma expanders**

	**LR**	**GEL**	**HES 200**	**HES 130**
Intrinsic viscosity (mPa.s)	0.673 ± 0.002	1.441 ± 0.015*	1.797 ± 0.013^#^*	1.529 ± 0.015^#^*^§^

Values are means ± SD.

*P < 0.05 versus LR, ^§^ *P* < 0.05 versus HES 200, ^#^ *P* < 0.05 versus GEL.

### Erythrocyte deformability

**Figure 2:**
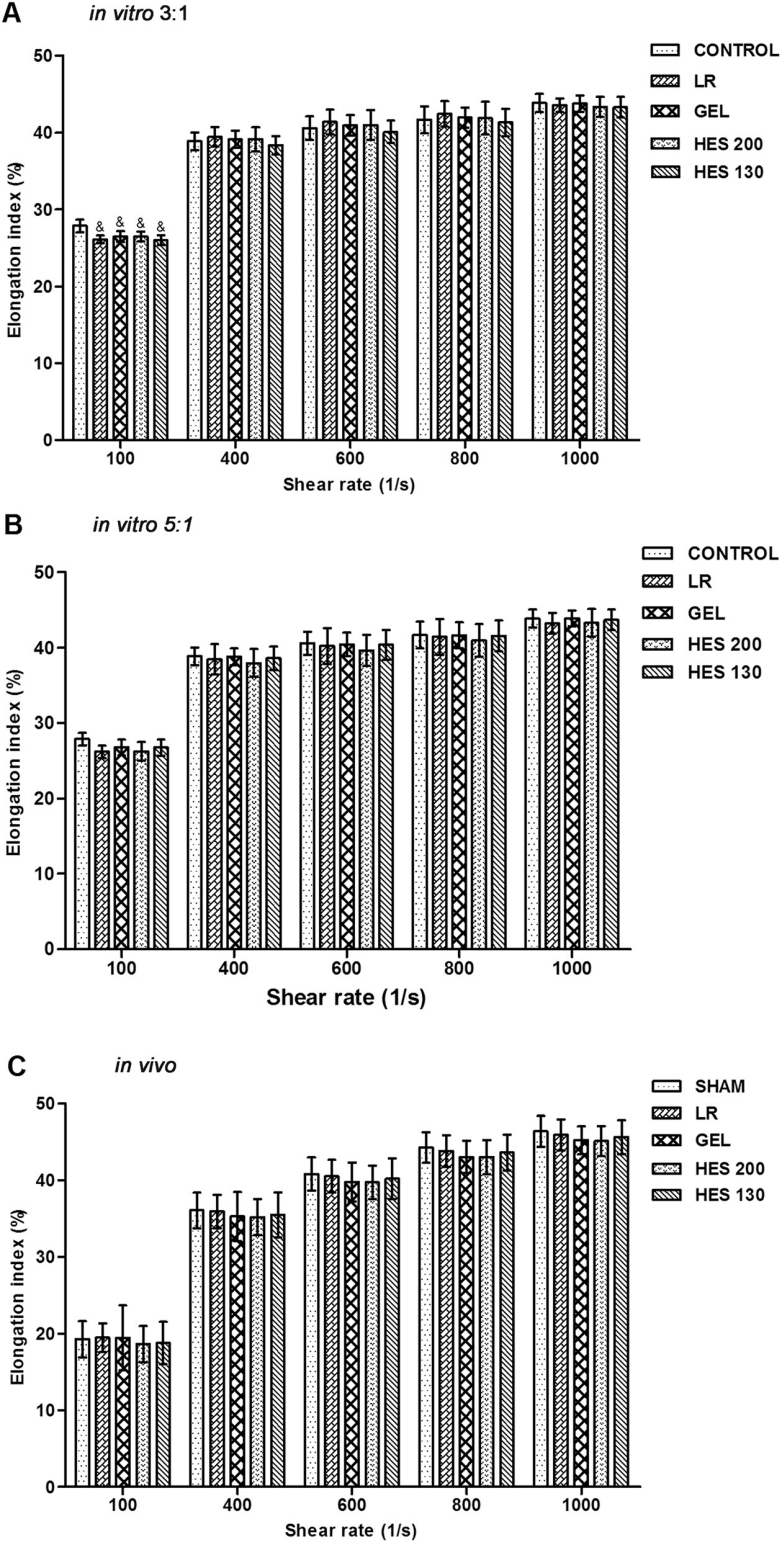
**Effects of various plasma expanders on erythrocyte deformability. (A)** Erythrocyte deformability at a ratio of 3:1 *in vitro* (*n* = 5). **(B)** Erythrocyte deformability at a ratio of 5:1 *in vitro* (*n* = 5). **(C)** Erythrocyte deformability *in vivo*. Data are plotted as the means ± SD. ^&^ *P* < 0.05 versus the control group.

For the ratio of 3:1, the erythrocyte deformability, as measured using EI, significantly decreased in the presence of the four plasma expanders at a shear rate of 100 s^−1^ (*P* < 0.05). However, the EI values at high rates (>100 s^−1^) did not differ significantly among the groups (Figure 2A). For the ratio of 5:1, there were no significant differences among the groups at the various shear rates (Figure 2B). In the *in vivo* study, the EI values at various shear rates for the study groups were not significantly different from those of the sham group (Figure 2C).

### Erythrocyte aggregation

**Figure 3:**
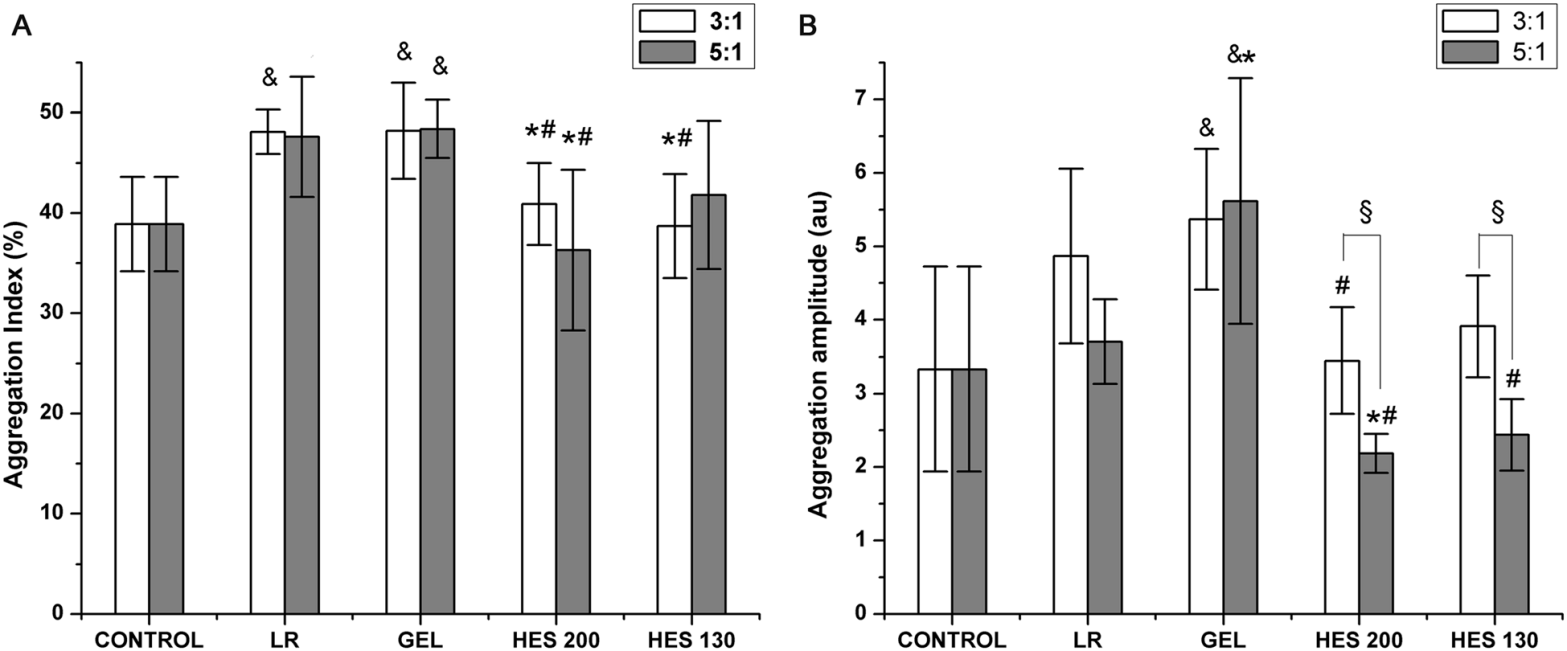
**Effects of various plasma expanders on erythrocyte aggregation** ***in vitro*** **. (A)** AI at various mixing ratios (*n* = 5). **(B)** AMP at various mixing ratios (*n* = 5). Data are plotted as the means ± SD. ^&^ *P* < 0.05 versus the control group; *P < 0.05 versus the LR group; ^#^ *P* < 0.05 versus the GEL group; ^§^ *P* < 0.05 versus the ratio of 3:1.

The *in vitro* erythrocyte aggregation measurements are presented in Figure 3. For the ratio of 3:1, GEL induced hyperaggregation by significantly increasing the AI and AMP compared to those of the control group. AI was significantly lower in the HES 130 and HES 200 groups compared to that of the GEL group (*P* < 0.05); AMP was significantly lower in the HES 200 group compared to that of the GEL group (*P* < 0.05). There were no significant differences among the HES 130, HES 200, and control groups with respect to AI and AMP (*P* > 0.05); AI was significantly lower in the HES 200 and HES 130 groups compared to that of the LR group (*P* < 0.05). For the ratio of 5:1, GEL induced a dramatic elevation in AI and AMP compared to those of the control group (*P* < 0.05). The AI and AMP were significantly lower in the HES 200 group compared to those in the GEL group (*P* < 0.05). There were no significant differences among the HES 130, HES 200, and control groups with respect to AI and AMP; AI was significantly lower in the HES 200 and HES 130 groups compared to that in the LR group (*P* < 0.05, Figure 3A, B). In addition, there were no differences for AI among the various ratios. The AMP values in the HES 130 and HES 200 groups for the ratio of 3:1 were significantly increased compared to those for the ratio of 5:1 (*P* < 0.05, Figure 3A, B).

**Figure 4:**
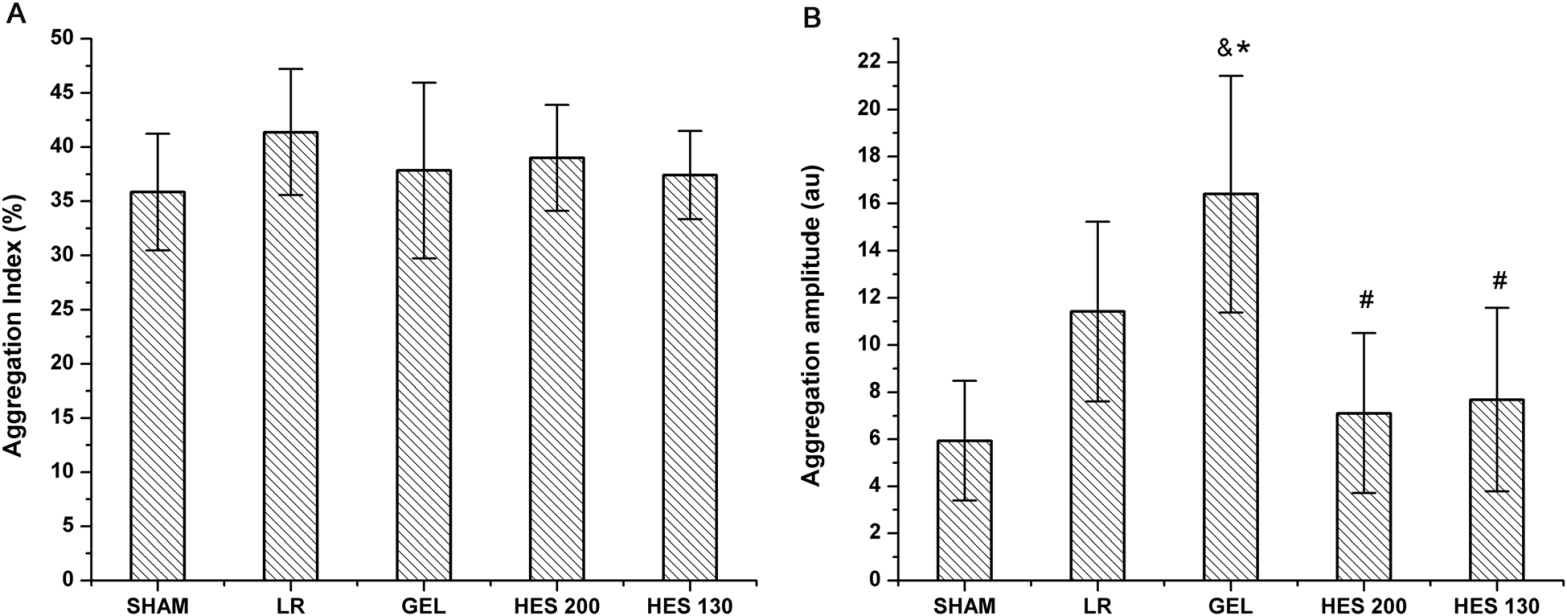
**Effects of various plasma expanders on erythrocyte aggregation (AI and AMP)** ***in vivo*** **. (A)** AI *in vivo*. **(B)** AMP *in vivo*. Data are plotted as the means ± SD. ^&^ *P* < 0.05 versus the sham group; *P < 0.05 versus the LR group; ^#^ *P* < 0.05 versus the GEL group.

There were no significant differences among the groups with respect to the AI values *in vivo* (Figure 4A). GEL induced a significant elevation in AMP compared to those of the LR and sham groups (*P* < 0.05). The AMP values in the HES 130 and HES 200 groups were significantly lower than that of the GEL group (*P* < 0.05). There were no significant differences among the HES 130, HES 200, LR, and sham groups with respect to AMP (Figure 4B).

## Discussion

Different fluids under clinical conditions have been found to cause different hemorheological effects, which may contribute to the persistence or deterioration of the physiological state [7,22]. The current study was conducted to investigate the influence of synthetic colloids (HES 130, HES 200, and GEL) and LR on plasma viscosity, erythrocyte aggregability, and erythrocyte deformability *in vitro* and in a rodent model of controlled hemorrhage *in vivo*.

The hemorheological parameters greatly influence blood circulation [23]. In rat spinotrapezius muscle, Kim et al. demonstrated that induction of erythrocyte aggregation using dextran 500 at a lower arterial pressure reduces the functional capillary density, which is an important indicator of microcirculatory dysfunction [24]. Enhanced erythrocyte aggregation may be associated with decreased wall shear stresses and results in the suppression of nitric oxide release, which plays a key role in the control of circulatory function [25,26]. Furthermore, O_2_ release from erythrocytes to tissues are physiologically important; erythrocyte aggregation is one of several important factors affecting O_2_ release in microvessels [27,28]. Restoring the rheological properties of the blood by enhancing the plasma viscosity is beneficial for maintaining the systemic and microhemodynamic parameters during a state of hypovolemic shock [29].

The main finding was the markedly different effects of the buffered and non-buffered solutions on red blood cell aggregation, both *in vitro* and in a rodent model of controlled hemorrhage *in vivo*. At the doses used in these experiments, we found that HES 130 and HES 200 did not change erythrocyte aggregation compared to the control. GEL had a potent hyperaggregating effect on rat erythrocyte. Erythrocyte aggregation in the GEL and LR groups was significantly higher compared to those in the HES 130 and HES 200 groups, according to the *in vitro* study. In the HS model, GEL markedly increased the aggregation amplitude compared to HES 130 and HES 200. However, there was no difference in the aggregation index among the groups. These results are not consistent with previous observations for HES 200 [6,13]. Earlier reports suggested the HES 200 may affect normal erythrocyte rheology. Henkelman et al. demonstrated that HES 200/0.5 enhanced erythrocyte aggregation *in vitro* using a laser-assisted optical rotational cell analyzer [14]. Differences in the concentration of colloid may account for these controversial results. In this study, erythrocyte aggregation was tested on erythrocytes suspended in 10% 200-kDa HES solutions. The final concentration of HES is higher than that of our present study (1% and 1.5%). It has been shown that the aggregation of erythrocytes was extended and accelerated by increasing the concentration of HES [13]. In contrast, our results for HES were consistent with a report of preoperative hemodilution [4]. The erythrocyte aggregation was unchanged with HES 200 in the study.

In this study, the ability of erythrocytes to form aggregates was measured by the aggregation amplitude and aggregation index. The prominent effect observed *in vitro* is in accordance with *in vivo* data for aggregation amplitude. However, the data for aggregation index in the *in vitro* study is not in accordance with the *in vivo* data. Our results revealed this incongruence between the *in vivo* and *in vitro* studies. The aggregation amplitude represents the extent of the aggregation. The overall aggregation behavior of the suspension is described by the aggregation index. The aggregation amplitude may be more sensitive than the aggregation index. It has been indicated that the aggregation amplitude is significantly higher in patients with slow coronary flow, whereas there was no significant difference between the groups with respect to the aggregation index [30]. In another study, Hazer et al. demonstrated the effect of pravastatin on hemorheological parameters in a rodent traumatic brain injury model. The aggregation amplitude first increased from the second day to the seventh day in the sham-operated group; however, the aggregation index remained stable on the seventh day. In the pravastatin group, the aggregation amplitude significantly increased on the 15th day, whereas the aggregation index remained stable on the 15th day [31]. This finding indicates that the aggregation amplitude should be taken into consideration when evaluating the erythrocyte aggregation, especially during the early stages of changes in the erythrocyte aggregation.

There have been few studies on colloid concentrations in plasma after hemorrhagic shock/resuscitation. The final concentrations (1% and 1.5%) for HES that were used in the present *in vitro* study were comparable to values observed in clinical studies. It was reported that patients undergoing orthopedic surgery have mean HES plasma concentrations of approximately 1% at the end of surgery [17]. During hemodilution therapy in patients with cerebrovascular diseases, the mean HES plasma peak concentration was 1.5% [18]. Therefore, the *in vitro* data we present are likely to reflect clinical conditions during large-dose colloid administration.

The plasma viscosity was significantly increased when GEL was used compared with HES 130 and HES 200, both *in vitro* and *in vivo*. However, the intrinsic viscosity of GEL was lower than those of HES 130 and HES 200. The various interactions between colloids and plasma proteins may account for this result. Previous studies have indicated that the solution viscosity increases with the rise of molecular mass of a given type of colloid at a constant concentration [32,33]. As mentioned above, gelatin has also been shown to interact specifically with plasma proteins, such as fibronectin [34], and may therefore enhance the formation of macromolecules, leading to increased plasma viscosity.

In our previous study, we compared the effects of different solutions including HES 130, HES 200, GEL, LR, normal saline, and Ringer acetate on hemorheology in an *in vitro* study and found there were no significant differences for hemorheological parameters between isotonic crystalloids. Therefore, we finally chose LR as the control in this study. In addition, the results *in vitro* are not directly comparable with *in vivo* coagulation in rats.

For the ratio of 3:1 *in vitro* study, LR was indicated to increase AI. There is no doubt that LR can decrease plasma protein concentrations and plasma viscosity. The decrease of plasma protein concentrations results in the inhibition of erythrocyte aggregation [35,36]. The result in this study implies that other factors also were involved in the increased erythrocyte aggregation. The divalent cations (Ca^+ +^) contained in LR are indicated to exacerbate the erythrocyte aggregation by decreasing the erythrocyte surface potential and electric double layer [37]. In addition, although it remains controversial, the lactate level was positively correlated with erythrocyte aggregation [38,39]. Consequently, the divalent cations and lactate may account for the increased erythrocyte aggregation.

A previous study has indicated that the pH levels can affect the erythrocyte aggregation [40]. There is a huge range from four different solutions (4.7–7.1). However, we found that the pH level of blood samples showed negligible changes when the samples mixed with four different solutions. It is inferred that the buffering capacity of the blood samples may prevent the pH levels from changing. The pH levels did not account for the difference of erythrocyte aggregation in this study.

The present study has limitations. First, the present *in vivo* study examined only a single time point, i.e., 2 h after treatment. Second, the results of blood gas and lactate concentration were absent. In addition, the volume of resuscitation was three times that of the blood withdrawal for LR in this study. The volume may hyperinfuse the rats, which may have an impact on the results.

## Conclusions

In summary, our study indicates that different synthetic colloids used for resuscitation produce various hemorheological effects, particularly with respect to erythrocyte aggregation. We also determined that GEL significantly accelerates the erythrocyte aggregation and significantly elevates the plasma viscosity compared to hydroxyethyl starch; furthermore, hydroxyethyl starch did not change the erythrocyte aggregation compared to that of the control group. In addition, *in vitro* hemorheological measurements are likely to provide hints for *in vivo* studies.
